# Surgery-related characteristics, efficacy, safety and surgical team satisfaction of three-dimensional heads-up system versus traditional microscopic equipment for various vitreoretinal diseases

**DOI:** 10.1007/s00417-022-05850-z

**Published:** 2022-10-10

**Authors:** Xin-yu Zhao, Qing Zhao, Ning-ning Li, Li-hui Meng, Wen-fei Zhang, Er-qian Wang, You-xin Chen

**Affiliations:** 1 Department of Ophthalmology, Peking Union Medical College Hospital, Chinese Academy of Medical Sciences, Beijing, 100730 China; 2 Key Laboratory of Ocular Fundus Diseases, Chinese Academy of Medical Sciences & Peking Union Medical College, Beijing, 100730 China; 3 Department of Operating Room, Peking Union Medical College Hospital, Chinese Academy of Medical Sciences, Beijing, 100730 China

**Keywords:** Three-dimensional heads-up surgery, Vitreoretinal surgery, Efficacy, Safety, Satisfaction

## Abstract

**Purpose:**

To compare the three-dimensional (3D) heads-up surgery with the traditional microscopic (TM) surgery for various vitreoretinal diseases.

**Methods:**

A medical record review of patients that underwent 3D heads-up or TM vitreoretinal surgeries was performed from May 2020 to October 2021 in this retrospective case–control study. Main outcome measures included surgery-related characteristics, efficacy, safety, and satisfaction feedback from the surgical team.

**Results:**

A total of 220 (47.6%) and 242 (52.4%) eyes were included in the 3D and TM groups, respectively. The 3D heads-up system significantly benefits delicate surgical steps, like the epiretinal membrane (ERM) peeling for ERM and internal limiting membrane peeling for idiopathic macular holes (*P* < 0.05). The 3D heads-up system could facilitate a significantly better visual outcome for pathologic myopic foveoschisis (*P* = 0.049), while no difference by TM surgery (*P* = 0.45). For the satisfaction feedback, the 3D heads-up system was rated significantly higher in most subscales and the overall score (*P* < 0.05). The surgeons’ ratings on operating accuracy and the first assistants’ rating on operating accuracy and operation cooperation were significantly higher in the TM group than in the 3D group (*P* < 0.05). Besides that, the 3D heads-up surgery was comparable with TM surgery in the surgery-related characteristics, choice of tamponades, postoperative VA, primary anatomic success, and perioperative complications (*P* > 0.05).

**Conclusion:**

The efficacy and safety of the 3D heads-up surgery were generally comparable to the TM surgery. The 3D heads-up system could significantly benefit delicate surgical steps and achieve better surgical team satisfaction.


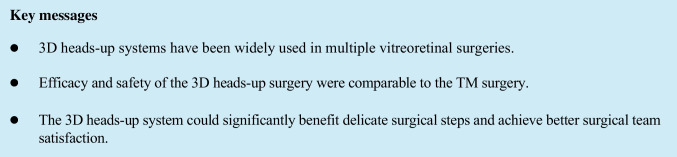


## Introduction

Three-dimensional (3D) heads-up surgery was first developed and applied in ophthalmic surgery in 2009, mostly in anterior segment surgery [1, 2]. In 2016, Eckardt et al. [3] firstly reported the application of the 3D heads-up system in more complicated vitrectomy surgery, increasing the popularity among ophthalmologists for treating vitreoretinal diseases. Nowadays, 3D heads-up systems have been widely used in multiple vitreoretinal surgeries, including macular membrane peeling for epiretinal membrane (ERM), repair of macular hole (MH) and rhegmatogenous retinal detachment (RRD), and vitrectomy for non-clearing vitreous hemorrhage (VH) and tractional retinal detachments (TRD). The current clinical or research- used 3D heads-up systems in ophthalmic surgeries included the Alcon NGENUITY® 3D Visualization System (Alcon Laboratories, Fort Worth, TX), the TrueVision Visualization System (Santa Barbara, CA), and the NCVideo3D system (NewComm, Beijing, China).

The 3D heads-up surgery system was reported to have multiple advantages over the traditional microscopic (TM) system, including high magnification performance, superior stereoscopic sensation, wide visual field, expanded depth of field, and reduced retinal phototoxicity, display image manipulation, improved ergonomics, and enhanced surgical team communication and education [3–9]. Previously reported disadvantages included the costly equipment, the learning curve required to use it efficiently, and the time latency between surgical interventions and their visualization [10, 11], while recent studies found that the time latency of the current 3D heads-up system may not jeopardize the surgical performance and outcome [12, 13].

However, several issues still needed to be settled. (1) Most previous studies evaluating the 3D heads-up system in vitreoretinal surgeries focused only on one single vitreoretinal disease [11, 14]. Studies with a larger sample size, multiple vitreoretinal diseases, and more comprehensive analysis were needed to better describe the merit and demerit of the 3D-heads-up system; (2) only a few studies have compared the outcomes of surgeries, for example, visual acuity (VA), primary anatomic success, and postoperative complications between surgeries using the 3D heads-up system and TM equipment, and their sample size was also limited. Thus, their conclusion might be unsolid [15, 16]; (3) whether 3D heads-up surgery was associated with longer surgical duration or longer learning curve remained controversial. Some studies reported 3D heads-up surgery with a longer learning curve and longer surgical duration, while other studies found no significant difference [16–18].

Our study aimed to investigate the surgery-related characteristics, efficacy, safety, and surgical team satisfaction feedback between the 3D heads-up surgery and TM surgery for common vitreoretinal diseases. The duration of specific surgical steps, visual outcomes, primary anatomic success rate, perioperative complications, and subjective assessment from the surgery team were compared in detail to obtain a more comprehensive description of the 3D heads-up surgery system and provide references for ophthalmologists.

## Methods

### Study design

**Fig. 1:**
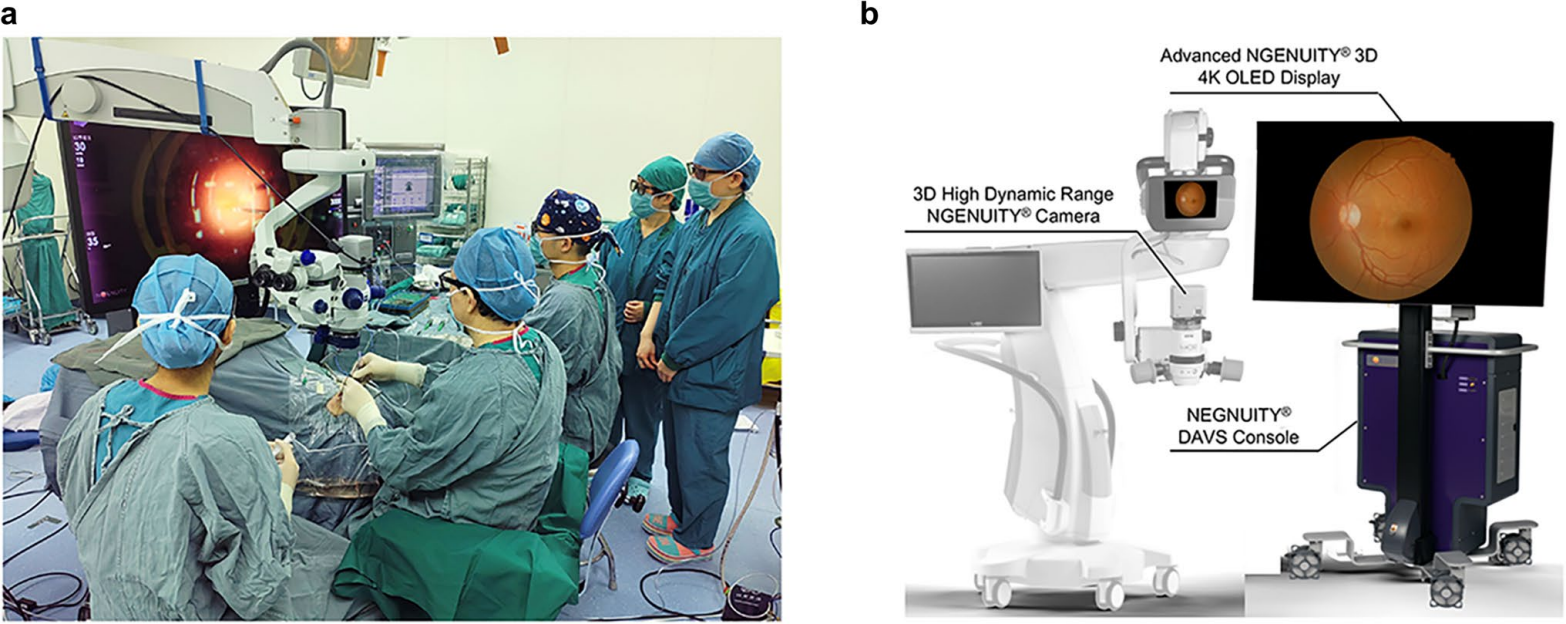
**a** The surgical team during the surgery using the 3D heads-up visualization system. Every member in the surgical team wore passive polarized 3D glasses and viewed the surgical field on the Advanced NGENUITY^®^ 3D 4 K OLED Display. The NGENUITY^®^ 3D Visualization System and the CONSTELLATION^®^ Vision System establish an Integrated Surgical Platform, which could monitor the real-time IOP, flow rate, and other surgical parameters. **b** The Alcon NGENUITY^®^ 3D Visualization System. Abbreviations: ILM = internal limiting membrane; IO*P* = intraocular pressure; 2D = two-dimensional; 3D = three-dimensional

A medical record review of patients who underwent vitreoretinal surgeries using the 3D heads-up system (3D group) or TM equipment (TM group) was performed from May 2020 to October 2021. All patients were examined and treated by the same surgeon (YXC) at the Ophthalmology Department of Peking Union Medical College Hospital (PUMCH) in Beijing, China. This retrospective study was approved by the Institutional Review Board/Ethics Committee of PUMCH (No. S-K1944) and was conducted following the tenets of the Declaration of Helsinki. Written informed consent was provided to each patient before the surgery. All the healthcare staff presented in Fig. 1a had given informed consent for publication.

### Inclusion and exclusion criteria

The following inclusion criteria were used: (1) patients underwent vitreoretinal surgeries for ERM, vitreomacular traction syndrome (VMT), VH, TRD, MH, RRD, pathologic myopic (PM) foveoschisis, silicone oil removal (SOR), and vitreous opacities using the 3D heads-up system or the TM equipment; (2) patients with detailed medical records and underwent comprehensive ophthalmologic examination including the Snellen best-corrected visual acuity (BCVA), intraocular pressure (IOP), axial lengths (AL), slit-lamp biomicroscopy, optical coherence tomography (OCT), and fundus photograph (FP); (3) a minimum follow-up period of 3 months after the surgery. The exclusion criteria were the following: (1) any other concomitant ocular diseases that could confound the results of the included vitreoretinal diseases; (2) patients with insufficient medical data or lost to follow-up. When both eyes of one patient were eligible, both eyes were included in the study.

### Surgical procedure

All surgeries were performed with the Alcon Constellation surgery system (Alcon Laboratories, Fort Worth, TX) by the same surgeon (YXC) with experience in vitreoretinal surgeries for more than 30 years. The TM group used the traditional microscopic system (OPMI-Lumera 700 with ReSight; Carl Zeiss Meditec AG; Jena, Germany), and the 3D group used Alcon NGENUITY^®^ 3D Visualization System (Alcon Laboratories, Fort Worth, TX). This 3D visualization system was mainly composed of the 3D High Dynamic Range NGENUITY^®^ Camera, advanced NGENUITY^®^ 3D 4 K OLED Display, and NGENUITY^®^ DAVS Console (see Fig. 1b). All patients underwent standard 23-gauge or 25-gauge three-port pars plana vitrectomy (PPV) under local retrobulbar anesthesia or general anesthesia. All pre-, peri-, and pos-toperative regimens were the same in these two groups. After the eyes were disinfected with 5% povidone-iodine and the conjunctiva was displaced by approximately 1–2 mm, trocar cannulas were inserted at a 20–30° angle into the conventional inferotemporal, superotemporal, and superonasal quadrants 3.5–4 mm posterior to the limbus. Surgical procedures vary according to the surgical indicators. Triamcinolone acetonide (TA), indocyanine green (ICG), liquid perfluorocarbon (C3F8), endodiathermy, retinotomy, and endolaser coagulation were applied as surgical adjuncts if necessary. The inverted internal limiting membrane (ILM) flap or the ILM insertion was applied in eyes with idiopathic MHs or PM-related MHs. The ILM around the fovea was peeled in the eyes with PM foveoschisis. Fluid-air exchange and tamponades of air, balanced salt solution (BSS), 10–14% C3F8, or silicone oil were performed based on the operating surgeon’s discretion when indicated.

### Data collection

Information extracted from the medical records of patients included age, gender, operative eye, AL, ocular and surgical history, diagnosis and surgical indicators, surgical procedures, choice of tamponades, pre- and pos-toperative Snellen BCVA, pre- and pos-toperative IOP, perioperative complications, general surgical duration, and duration of specific steps (e.g., ILM peeling). Twenty surgeries were randomly selected in a 1:1 ratio from the 3D group and the TM group by an independent analyzer. The satisfaction questionnaires evaluating surgery-related characteristics (e.g., resolution, magnification, depth of the field) and general satisfaction feedback to the surgical system were requested to be finished by the surgeon, first assistant, instrument nurses, and visitors immediately after the surgery. The postoperative follow-up was scheduled at approximately 1 week, 1 month, and 3 months, with the measurement of Snellen BCVA, IOP, FP, etc.

### Outcome measures

The main outcome measures included Snellen BCVA, primary anatomic success, general surgical duration, duration of specific steps, perioperative complications, and satisfaction feedback from the surgical team. The Snellen BCVA was converted to the logarithm of the minimum angle of resolution (logMAR) equivalents for statistical analysis [19]. No light perception (NLP) was set at 2.90 logMAR, light perception (LP) at 2.60 logMAR, hand movements (HM) at 2.30 logMAR, and fingers counting (FC) at 1.85 logMAR [20]. The definition of anatomic success varied according to the surgical indicators and was evaluated by two retinal specialists (XYZ and QZ). The primary anatomic success was defined as complete removal of ERM for eyes with ERM, relieving of VMT for VMT, clearance of VH and vitreous opacities for VH, reattachment of the retina for TRD and RRD, closure of MH for MH, recovery of PM foveoschisis for PM foveoschisis, removal of silicone oil and the attachment of retina for SOR, and disappearance of vitreous opacities for vitreous opacities. The duration of ILM peeling was defined as from the starting of ICG injection to the finishing of ILM peeling. The general surgical duration was defined as starting the trocar insertion to finishing the wound sealing. Ocular hypertension was defined as IOP ≥ 21 mmHg during the postoperative follow-up.

### Statistical analysis

Data were analyzed by univariable analysis by comparing each aforementioned parameter between the 3D group and the TM group. Analyses were performed independently for each subgroup of vitreoretinal disease. Continuous variables were summarized as mean ± standard deviation (SD) and categorical data were presented as frequency (percentages). The independent *t*-test and two-tailed, paired *t*-test were used to evaluate comparative statistical analyses. The chi-squared test or Fisher’s exact test was used to examine categorical variables. All statistical analyses were performed with Stata SE 12.0 software (StataCorp, College Station, TX, USA). The two-tailed *p*-value < 0.05 was considered statistically significant.

## Results

A total of 426 patients and 462 eyes were finally enrolled, of which 220 (47.6%) and 242 (52.4%) eyes were included in the 3D and TM groups, respectively. Among the included patients, 218 (51.2%) were female and 208 (48.8%) were male, with a mean age of 55.00 ± 14.36 years. Age, AL, and pseudophakic eye showed no statistical differences (*P* > 0.05) between the 3D and TM groups (see Table 1).

**Table 1:** Baseline demographics of patients in the 3D group and the TM group

Surgical indicators	Patients (eyes)	Group Size (eyes)		Age (mean ﻿ ± SD, years)			AL (mean ﻿ ± SD, mm)			Pseudophakic eye (*n*, %)		
		TM	3D	TM	3D	*P*	TM	3D	*P*	TM	3D	*P*
Idiopathic ERM	66(70)	39	31	64.35 ﻿ ± 14.18	61.08 ﻿ ± 14.13	0.34	23.17 ﻿ ± 1.21	23.66 ﻿ ± 1.26	0.10	7(17.9)	9(29.0)	0.27
VMT	12(12)	5	7	61.25 ﻿ ± 13.67	64.67 ﻿ ± 12.79	0.67	24.67 ﻿ ± 1.27	23.47 ﻿ ± 1.05	0.10	0(0)	1(14.3)	0.86
VH												
With TRD	42(47)	25	22	48.23 ﻿ ± 12.46	46.45 ﻿ ± 12.07	0.51	22.88 ﻿ ± 1.43	23.58 ﻿ ± 1.72	0.13	8(32.0)	9(40.9)	0.53
Without TRD	80(85)	51	34	57.19 ﻿ ± 11.87	55.58 ﻿ ± 12.38	0.55	23.74 ﻿ ± 0.99	23.08 ﻿ ± 2.26	0.07	9(17.6)	11(32.4)	0.12
MH												
Idiopathic MH	32(34)	14	20	57.75 ﻿ ± 14.28	62.47 ﻿ ± 12.56	0.31	23.79 ﻿ ± 1.48	23.63 ﻿ ± 1.39	0.75	2(14.3)	4(20.0)	0.98
PM-related MH	24(29)	12	17	60.57 ﻿ ± 10.91	57.13 ﻿ ± 10.36	0.40	28.53 ﻿ ± 2.18	28.89 ﻿ ± 2.21	0.67	2(16.7)	2(11.8)	0.87
RRD												
Primary RRD	43(45)	25	20	53.83 ﻿ ± 12.25	48.67 ﻿ ± 13.28	0.18	25.56 ﻿ ± 3.44	25.58 ﻿ ± 4.57	0.99	3(12.0)	1(5.0)	0.77
PVR-related RRD	29(33)	19	14	45.43 ﻿ ± 15.39	50.90 ﻿ ± 16.05	0.33	24.69 ﻿ ± 2.75	23.88 ﻿ ± 2.99	0.43	4(21.1)	6(42.9)	0.18
PM foveoschisis	13(13)	4	9	49.75 ﻿ ± 16.52	52.75 ﻿ ± 13.75	0.74	27.52 ﻿ ± 2.14	28.08 ﻿ ± 1.88	0.64	0(0)	0(0)	NA
SOR												
For RRD	34(34)	14	20	55.27 ﻿ ± 14.19	51.32 ﻿ ± 14.71	0.44	25.76 ﻿ ± 3.82	25.81 ﻿ ± 4.23	0.97	4(28.5)	7(35.0)	0.98
For TRD	47(52)	30	22	46.89 ﻿ ± 16.64	50.09 ﻿ ± 14.83	0.48	24.69 ﻿ ± 2.75	23.88 ﻿ ± 2.99	0.32	7(23.3)	5(22.7)	0.96
Vitreous opacitie**s**	4(8)	4	4	49.23 ﻿ ± 15.91	45.00 ﻿ ± 20.79	0.76	32.44 ﻿ ± 2.51	34.17 ﻿ ± 3.99	0.49	0(0)	0 0)	NA
All	426(462)	242	220	54.79 ﻿ ± 14.19	54.32 ﻿ ± 14.54	0.73	24.52 ﻿ ± 2.28	24.74 ﻿ ± 2.63	0.34	46(19.0)	55(25.0)	0.12

*AL* axial length, *ERM* epiretinal membrane, *MH* macular hole, *PM* pathologic myopic, *PVR* proliferative vitreoretinopathy, *RRD* rhegmatogenous retinal detachment, *SD* standard deviation, *SOR* silicone oil removal, *TM* traditional microscopic, *TRD* tractional retinal detachments, *3D* three-dimensional, *VH* vitreous hemorrhage, *VMT* vitreomacular traction syndrome

The duration of ERM or ILM peeling for eyes with ERM and idiopathic MH was significantly shorter in the 3D group than in the TM group (ERM: 6.12 ± 2.45 versus 9.55 ± 5.34, *P* < 0.01; idiopathic MH: 6.03 ± 2.12 versus 9.01 ± 4.06, *P* = 0.01). Compared with the TM group, the 3D group was associated with significantly shorter general surgical duration for eyes with ERM (20.14 ± 6.72 versus 24.81 ± 8.62, *P* = 0.02) and idiopathic MH (22.13 ± 6.58 versus 26.75 ± 5.94, *P* = 0.04). No significant difference existed in the duration of complete vitrectomy and proliferating membrane peeling, the choice of tamponades, and the incidence of iatrogenic retinal breaks between the 3D group and TM group (*P* > 0.05) (see Table 2).

**Table 2:** Surgical duration and the choice of tamponades in the 3D group and the TM group

	TM	3D	*P*
Duration of ILM peeling (mean ﻿ ± SD, min)			
ERM	9.55 ± 5.34	6.12 ± 2.45	< 0.01*
VMT	8.69 ± 4.29	5.99 ± 3.79	0.28
Idiopathic MH	9.01 ± 4.06	6.03 ± 2.12	0.01*
PM-related MH	15.74 ± 7.93	12.97 ± 5.83	0.29
PM foveoschisis	16.74 ± 8.41	12.89 ± 6.74	0.40
Duration of complete vitrectomy (mean ﻿ ± SD, min)			
VH with TRD	21.14 ± 14.84	19.31 ± 8.19	0.61
VH without TRD	14.33 ± 3.32	15.57 ± 5.88	0.22
Primary RRD	16.49 ± 6.85	17.71 ± 7.42	0.57
PVR-related RRD	23.82 ± 15.69	22.37 ± 14.28	0.79
Duration of proliferating membrane peeling (mean ﻿ ± SD, min)			
VH with TRD	22.98 ± 15.09	25.71 ± 16.27	0.55
PVR-related RRD	24.15 ± 14.33	22.62 ± 13.11	0.76
General surgical duration (mean ﻿ ± SD, min)			
ERM	24.81 ± 8.62	20.14 ± 6.72	0.02*
VH			
With TRD	54.98 ± 24.92	49.04 ± 26.83	0.44
Without TRD	37.13 ± 22.05	35.62 ± 19.28	0.75
MH			
Idiopathic MH	26.75 ± 5.94	22.13 ± 6.58	0.04*
PM-related MH	29.72 ± 15.89	27.72 ± 9.92	0.68
RRD			
Primary RRD	37.41 ± 8.77	36.51 ± 9.29	0.74
PVR-related RRD	54.74 ± 21.07	48.55 ± 25.01	0.45
PM foveoschisis	34.21 ± 12.21	32.11 ± 9.18	0.74
SOR			
For RRD	16.67 ± 8.14	16.09 ± 5.22	0.80
For TRD	25.45 ± 14.39	26.88 ± 15.98	0.74
Vitreous opacities	12.88 ± 3.31	13.64 ± 4.27	0.79
Choice of tamponades (*n*, %)	39	31	
ERM			
Air	6(15.4)	5(16.1)	
BSS	30(76.9)	25(80.6)	
C3F8	3(7.7)	1(3.2)	
Silicone oil	0(0)	0(0)	0.17
VMT	5	7	
Air	1(20.0)	2(28.6)	
BSS	4(80.0)	5(71.4)	
C3F8	0(0)	0(0)	
Silicone oil	0(0)	0(0)	0.74
VH with TRD	25	22	
Air	0(0)	0(0)	
BSS	0(0)	0(0)	
C3F8	0(0)	0(0)	
Silicone oil	25(100)	22(100)	NA
VH without TRD	51	34	
Air	10(19.6)	6(17.6)	
BSS	35(68.6)	24(70.6)	
C3F8	6(11.8)	4(11.8)	
Silicone oil	0(0)	0(0)	0.97
Idiopahtic MH	14	20	
Air	10(71.4)	16(80.0)	
C3F8	4(29)	4(20.0)	
Silicone oil	0(0)	0(0)	0.56
PM-related MH	12	17	
Air	0(0)	0(0)	
C3F8	9(75.0)	15(88.2)	
Silicone oil	3(25.0)	2(11.8)	0.35
PM foveoschisis	4	9	
C3F8	2(50.0)	5(55.6)	
Silicone oil	2(50.0)	4(44.4)	0.85
Primary RRD	25	20	
C3F8	11(44.0)	9(45.0)	
Silicone oil	14(56.0)	11(55.0)	0.95
PVR-related RRD	19	14	
C3F8	6(31.5)	2(14.3)	
Silicone oil	13(68.4)	12(85.7)	
Incidence of iatrogenic retinal breaks (*n*, %)	4(1.6)	6(2.7)	0.11

^*^P < 0.05

*BSS* balanced salt solution, *C3F8* perfluoropropane, *ERM* epiretinal membrane, *ILM* internal limiting membrane, *MH* macular hole, *NA* not available, *PM* pathologic myopic, *PVR* proliferative vitreoretinopathy, *RRD* rhegmatogenous retinal detachment, *SD* standard deviation, *SOR* silicone oil removal, *TM* traditional microscopic, *TRD* tractional retinal detachments, *3D* three-dimensional, *VH* vitreous hemorrhage, *VMT* vitreomacular traction syndrome

For eyes with idiopathic ERM, VH, idiopathic MH, RRD, and SOR, postoperative VA improved significantly compared with the preoperative VA both in the 3D group and the TM group (*P* < 0.05). For eyes with PM foveoschisis, significant postoperative VA improvement was noticed in the 3D group (0.57 ± 0.38 versus 1.00 ± 0.47, *P* = 0.049) but not in the TM group (0.63 ± 0.55 versus 0.97 ± 0.63, *P* = 0.45). No significant difference in preoperative VA, postoperative VA, primary anatomic success rate, and perioperative complications between the 3D group and the TM group (*P* > 0.05) (see Table 3).

**Table 3:** Visual acuity, anatomic success, and perioperative complications in the 3D group and the TM group

	Preoperative BCVA			Postoperative BCVA			*P* (Pre-versus Post-)		Primary anatomic success rate (*n*,%)			Complications											
												Ocular hypertension (*n*,%)			VH (*n*,%)			RD (*n*,%)			MH (*n*,%)		
	TM	3D	*P*	TM	3D	*P*	TM	3D	TM	3D	*P*	TM	3D	*P*	TM	3D	*P*	TM	3D	*P*	TM	3D	*P*
Idiopathic ERM (39/31)	0.78 ﻿ ± 0.61	0.81 ﻿ ± 0.57	0.84	0.45 ﻿ ± 0.40	0.48 ﻿ ± 0.59	0.80	0.01*	0.03*	39(100)	31(100)	NA	0(0)	2(6.5)	0.38	0(0)	0(0)	NA	0(0)	0(0)	NA	0(0)	0(0)	NA
VMT(5/7)	0.95 ﻿ ± 0.51	0.91 ﻿ ± 0.68	0.91	0.56 ﻿ ± 0.38	0.59 ﻿ ± 0.48	0.91	0.21	0.33	5(100)	7(100)	NA	1(20.0)	2(28.6)	0.74	0(0)	0(0)	NA	0(0)	0(0)	NA	0(0)	0(0)	NA
VH																							
With TRD (25/22)	1.66 ﻿ ± 0.64	1.77 ﻿ ± 0.62	0.55	1.02﻿ ± 0.55	1.07 ﻿ ± 0.83	0.81	< 0.01*	< 0.01*	20(80.0)	18(81.8)	0.83	5(20)	4(18.2)	0.83	2(8.0)	2(9.1)	0.70	3(12.0)	2(9.1)	0.88	0(0)	0(0)	NA
Without TRD (51/34)	1.61 ﻿ ± 0.75	1.65 ﻿ ± 0.76	0.81	0.39 ﻿ ± 0.62	0.31 ± 0.79	0.60	< 0.01*	< 0.01*	48(94.1)	33(97.1)	0.92	5(9.8)	5(14.7)	0.73	3(5.9)	1(2.9)	0.92	0(0)	0(0)	NA	0(0)	0(0)	NA
MH																							
Idiopathic MH (14/20)	1.02 ﻿ ± 0.30	0.95 ﻿ ± 0.35	0.55	0.74 ﻿ ± 0.32	0.69 ﻿ ± 0.28	0.63	0.02*	0.01*	12(85.7)	20(100)	0.32	2(14.3)	(0)	0.32	0(0)	0(0)	NA	0(0)	0(0)	NA	2(14.3)	0(0)	0.32
PM-related MH (12/17)	1.17 ﻿ ± 0.69	1.12﻿ ± 0.53	0.83	0.97 ﻿ ± 0.92	0.95 ﻿ ± 0.68	0.95	0.55	0.42	9(75.0)	15(88.2)	0.67	3(25.0)	4(23.5)	0.73	0(0)	0(0)	NA	0(0)	0(0)	NA	3(25.0)	2(11.8)	0.67
RRD																							
Primary RRD (25/20)	1.49 ﻿ ± 0.77	1.44﻿ ± 0.68	0.82	0.98 ± 0.74	0.91﻿ ± 0.54	0.73	0.02*	0.01*	22(88.0)	18(90.0)	0.79	7(28.0)	3(15.0)	0.50	0(0)	0(0)	NA	3(12.0)	2(10.0)	0.79	0(0)	0(0)	NA
PVR-related RRD (19/14)	1.62 ﻿ ± 0.73	1.65﻿ ± 0.58	0.90	1.10﻿ ± 0.59	1.09 ﻿ ± 0.61	0.94	0.02*	0.02*	14(73.6)	11(78.6)	0.93	6(31.6)	2(14.3)	0.46	0(0)	0(0)	NA	5(26.3)	3(21.4)	0.93	0(0)	0(0)	NA
PM foveoschisis (4/9)	0.97 ﻿ ± 0.63	1.00 ﻿ ± 0.47	0.93	0.63 ﻿ ± 0.55	0.57 ﻿ ± 0.38	0.82	0.45	0.049*	3(75.0)	8(88.9)	0.85	1(25.0)	2(22.2)	0.55	0(0)	0(0)	NA	0(0)	0(0)	NA	0(0)	0(0)	NA
SOR																							
For RRD (14/20)	1.09 ﻿ ± 0.56	1.13 ﻿ ± 0.64	0.85	0.69 ﻿ ± 0.45	0.66 ﻿ ± 0.49	0.86	0.047*	0.01*	12(85.7)	19(95.0)	0.75	1(7.1)	1(5.0)	0.63	0(0)	0(0)	NA	2(14.3)	1(5.0)	0.75	0(0)	0(0)	NA
For TRD (30/22)	1.21 ﻿ ± 0.62	1.18﻿ ± 0.57	0.86	0.80 ﻿ ± 0.51	0.79 ﻿ ± 0.62	0.95	0.01*	0.045*	24(80.0)	18(81.8)	0.87	2(6.7)	1(4.5)	0.78	0(0)	0(0)	NA	6(20.0)	4(18.2)	0.85	0(0)	0(0)	NA
Vitreous opacities (4/4)	0.14 ﻿ ± 0.23	0.11 ﻿ ± 0.19	0.85	0.10 ﻿ ± 0.20	0.09 ﻿ ± 0.29	0.96	0.80	0.91	4(100)	4(100)	NA	0(0)	0(0)	NA	0(0)	0(0)	NA	0(0)	0(0)	NA	0(0)	0(0)	NA
All (242/220)	1.29 ﻿ ± 0.66	1.31 ﻿ ± 0.62	0.74	0.70 ﻿ ± 0.55	0.69 ﻿ ± 0.60	0.85	< 0.01*	< 0.01*	212(87.6)	202(91.8)	0.14	33(13.6)	26(11.8)	0.56	5(2.1)	3(1.4)	0.83	19(7.9)	12(5.5)	0.30	5(2.1)	2(0.9)	0.53

^*^P < 0.05

*BCVA* best-corrected visual acuity, *ERM* epiretinal membrane, *MH* macular hole, *NA* not available, *PM* pathologic myopic, *PVR* proliferative vitreoretinopathy, *RD* retinal detachment, *RRD* rhegmatogenous retinal detachment, *SOR* silicone oil removal, *TM* traditional microscopic, *TRD* tractional retinal detachments, *3D* three-dimensional, *VH* vitreous hemorrhage, *VMT* vitreomacular traction syndrome

In general, satisfaction feedback to the surgical system, the 3D heads-up system was rated significantly higher in most of the subscales (*P* < 0.05) and the overall score (212.48 ± 18.52 versus 160.17 ± 25.32, *P* < 0.01). The surgeons’ rating on instrument adjustment was comparable in these two groups (8.89 ± 1.30 versus 9.15 ± 0.62, *P* = 0.58). The TM group was rated significantly higher in the operating accuracy of the surgeon (9.56 ± 0.53 versus 8.22 ± 1.30, *P* = 0.01) and the operating accuracy (9.28 ± 0.80 versus 5.12 ± 2.21, *P* < 0.01) and operation cooperation (9.34 ± 0.71 versus 6.06 ± 2.43, *P* < 0.01) of the first assistant (see Table 4).

**Table 4:** General satisfaction feedback to the surgical system in the 3D group and the TM group

		TM	3D	*P*
Surgeon				
	Resolution of the lesion	7.44 ± 1.51	9.56﻿ ± 0.73	< 0.01*
	Magnification	6.33 ± 1.22	9.44 ± 0.73	< 0.01*
	Depth of Field	7.78 ± 0.97	9.00 ± 0.71	< 0.01*
	Operating accuracy	9.56 ± 0.53	8.22 ± 1.30	0.01*
	Comfort level	6.00 ± 1.12	9.44 ± 0.73	< 0.01*
	Instrument adjustment	9.15 ± 0.62	8.89 ± 1.30	0.58
	Operation cooperation	5.81 ± 1.78	9.51 ± 0.72	< 0.01*
	General satisfaction	8.12 ± 1.59	9.37 ± 0.88	0.04*
First assistant				
	Resolution of the lesion	6.89 ± 1.76	9.12 ± 0.82	< 0.01*
	Magnification	5.17 ± 2.02	9.15 ± 0.81	< 0.01*
	Depth of Field	7.41 ± 1.12	8.91 ± 0.95	< 0.01*
	Operating accuracy	9.28 ﻿ ± 0.80	5.12 ± 2.21	< 0.01*
	Operation cooperation	9.34 ± 0.71	6.06 ± 2.43	< 0.01*
	Comfort level	5.87 ± 1.76	9.30 ± 0.68	< 0.01*
	General satisfaction	7.25 ± 1.69	9.21 ± 0.81	< 0.01*
Instrument nurses				
	Understanding of surgical process	5.05 ± 2.15	9.00 ± 0.75	< 0.01*
	Instrument preparation	5.75 ± 1.84	9.10 ± 0.78	< 0.01*
	Active operation cooperation	6.02 ± 1.68	9.33 ± 0.66	< 0.01*
	Comfort level	5.00 ± 2.15	9.25 ± 0.72	< 0.01*
	General satisfaction	6.50 ± 1.75	9.13 ± 0.68	< 0.01*
Visitor				
	Understanding of surgical process	4.82 ± 2.65	9.25 ± 0.75	< 0.01*
	Resolution of the lesion	4.13 ± 2.71	9.31 ± 0.66	< 0.01*
	Magnification	3.75 ± 2.89	9.45 ± 0.62	< 0.01*
	Comfort level	3.50 ± 2.20	8.52 ± 1.29	< 0.01*
	General satisfaction	4.25 ± 2.55	9.40 ± 0.58	< 0.01*
Overall	Overall score	160.17 ± 25.32	212.48 ± 18.52	< 0.01*

^*^P < 0.05

*TM* traditional microscopic, *3D* three-dimensional

## Discussion

Our study evaluated the surgery-related characteristics, surgical outcomes, perioperative complications, and surgical team satisfaction between the 3D heads-up surgery system and the TM surgery for multiple vitreoretinal diseases. The results suggested that the 3D heads-up surgery system could significantly benefit in some delicate surgical steps, like ERM peeling for ERM and ILM peeling for idiopathic MH. The 3D heads-up system could facilitate a significantly better visual outcome for PM foveoschisis, while no difference existed with TM surgery. For the general satisfaction feedback, the 3D heads-up system was rated significantly higher in most of the subscales and the overall score, while the surgeons’ rating on operating accuracy, as well as the first assistants’ rating on operating accuracy and operation cooperation, were significantly higher in the TM group than the 3D group. Apart from the aforementioned findings, the 3D heads-up surgery system was as effective and safe as TM surgery in regards to the surgery-related characteristics, choice of tamponades, postoperative VA, primary anatomic success, and perioperative complications.

In our study, the duration of ILM peeling was significantly shorter in the 3D group than in the TM group for eyes with ERM or idiopathic MH. The possible reason could be that the 3D heads-up surgery has the advantage of high image magnification at a wider visual field compared with the TM system [21], the OPMI-Lumera 700 with ReSight, which enables surgeons to view the fine structures of the retina and then perform the membrane peeling more precisely. For surgical steps with less request of precision, such as complete vitrectomy, the duration was comparable between the 3D heads-up surgery and the TM surgery. This suggested that the advantage of the 3D heads-up system was more obvious when performing surgical steps of high precision [10, 21]. However, the inferiority of the TM system in handling precise surgical steps might also be associated with the specific TM operating system. Further research with other TM viewing operating systems was expected to compare the ability to handle precise surgical steps of the 3D heads-up system and the TM system.

Previous studies reported difficulties in controlling the depth of surgical operation using the 3D heads-up system. This might induce intraoperative complications like iatrogenic retinal breaks and require changing intraocular tamponades. Piccirillo et al. [11] reported the occurrence of 3 iatrogenic macular soft contusions in 10 procedures using the 3D heads-up system for vitreoretinal surgery, with no major retinal hemorrhages occurring. Our study found no difference in the incidence of iatrogenic retinal breaks or the choice of vitreous tamponades between the TM group and 3D group, and no differences existed in the occurrence of perioperative complications, including ocular hypertension, VH, RD, and MH. This indicated that the safety of the 3D heads-up surgery is comparable to the TM surgery [14].

The 3D heads-up surgery was comparable to the TM surgery in postoperative VA and primary anatomic success rate, which was in accordance with the previous studies [3, 10, 11, 16, 18, 21–23]. Although the 3D heads-up system could significantly shorten the duration of ILM peeling for eyes with ERM or idiopathic MH, no significant difference existed in the postoperative VA. However, the postoperative VA for eyes with PM foveoschisis significantly improved in the 3D group but not in the TM group. This indicated that the higher resolution of the 3D heads-up system enables more precise operations and better releasing of the retina, therefore promoting the recovery of the PM foveoschisis and the rehabilitation of visual function. The possible bias might exist due to the limited sample size of PM foveoschisis, and this finding should be confirmed in further studies.

In TM surgery, only the surgeon can have a high-grade stereo view of the surgical field, while the remaining observers (e.g. first assistant, instrument nurses, and visitors) could not appreciate the depth of field and the 3D view necessary for fine operations. However, by viewing a larger high-resolution screen of the 3D heads-up system, all members of the surgical team present can have access to the same live surgical image just as the surgeon. This provides significant improvements in operation cooperation and achieves additional pedagogical advantages [13, 24]. In this light, the surgeon could teach more easily and allow students or trainee surgeons to operate by reducing their installation time. In previous studies, surgeons and residents have rated the 3D system with improved ergonomics over TM [9]. The TM equipment was associated with more complaints of musculoskeletal pain because of the prolonged static unnatural neck-bent positions [25]. In contrast, by wearing polarized 3D glasses and viewing the surgical field on the 3D monitor without looking through microscope eyepieces in the neck-bent position, surgeons could turn their heads up through the 3D heads-up surgery system. Results obtained from the questionnaire designed ourselves showed that the 3D heads-up system was favored over the TM system in all subscales by instrument nurses and visitors. However, the first assistant rated lower scores in the subscale of “Operation cooperation” when using the 3D heads-up system, which was in accordance with the previously published study [7]. Rizzo et al. [7] evaluated the perceptions of the surgical team to the 3D surgical viewing system and recorded the first assistants’ dissatisfaction with the question “second surgeon’s comfort during surgery.” The first assistant has to rotate his head uncomfortably to look at the screen. The first assistant has to bear the inconsistency between the direction of the screen and the direction of the surgical steps such as trimming and pressuring, further increasing the difficulties of the surgery. However, this disadvantage could be overcome if the first assistant performed these surgical steps using the assistant microscope.

We summarized five keys to the satisfactory surgical experience of using the 3D heads-up system. Firstly, set the aperture at 30% to get the best brightness and depth of field. Secondly, set the white balance to eliminate chromatic aberration and restore the original color. Thirdly, set the display screen 1–1.2 m away from the surgeon’s eye level to achieve the best resolution and 3D effect. And the center of the display screen should be set perpendicular to the surgeon’s visual axis to reduce the double image of peripheral images. Fourthly, the focus should be adjusted occasionally. For the anterior segment, zoom in to the maximum, fine the focus on the iris to get a clear image, and then zoom out to the suitable image size. For the posterior segment, ensure that the non-contact lens is in the shortest position and is placed well. In the targeted surgical area, zoom in to the maximum, focus to the clearest, and then zoom out to the appropriate image size. Finally, the surgical field should fill the display screen, which could bring a better depth of field, resolution, and contrast, making the image more immersive.

Several limitations of our study should be noted. Firstly, OCT and FP were not assigned to every patient in each follow-up, and some information could not be extracted due to the retrospective nature of this study. Secondly, the last follow-up period was only 3 months which might have underestimated the rate of perioperative complications and overestimated the primary anatomic success rate. Thirdly, the detailed satisfaction feedback for surgically treating specific types of vitreoretinal disease was not investigated in our study because of the limited amount of feedback. Further studies with a prospective design and a longer follow-up period are needed to confirm our findings.

In summary, the efficacy and safety of the 3D heads-up surgery were generally comparable to the TM surgery. The 3D system could significantly benefit delicate surgical steps. Moreover, the 3D heads-up surgery performed better in the surgical team satisfaction. The 3D heads-up system, with all these advantages, could be recommended for patients with vitreoretinal diseases, especially those with ERM or idiopathic MH.
